# Patients treated outside ICUs

**DOI:** 10.1186/cc14675

**Published:** 2015-09-28

**Authors:** Ana Vitoria CS Gasparine, Cintia MC Grion, Claudia MDM Carrilho, Fabiane Urizzi, Joao Paulo M Favoreto, Patricia R Pêras, Patricia S Moya, Raquel Mireski

**Affiliations:** 1 Universidade Estadual de Londrina, Vila Operária, Paraná, Brazil

## Introduction

Intensive care beds are scarce resources, particularly in developing countries. For this reason it is not uncommon for critically ill patients to be treated in emergency rooms or in hospital wards due to unavailability of intensive care beds.

## Objective

To describe direct costs of treatment of critically ill patients treated outside ICUs due to a full unit.

## Methods

Prospective cohort study of critically ill patients treated outside the ICU by the ward health care staff with daily intensivist physician consultation in a university hospital during a 6-month period. All consecutive patients who were treated outside the ICU due to a full unit during the period of February 2012-February 2013 were included in the study. Patients were followed daily until they were transferred to an ICU bed or the request for intensive care was cancelled. Clinical and demographic data were collected to characterize the study sample and data to calculate costs using the bottom-up method were collected and grouped into four categories: clinical support, consumable items, human resources and hospital taxes. The Brazilian Medical Association (AMB) price index for medical procedures and the BRASINDICE price index for medications, solutions and hospital consumables were used to calculated costs.

## Results

One hundred and fifty-one patients were included in the study. Median age was 64 (interquartile range (ITQ): 49-72) years and 55% were male. Median APACHE II score was 23 (ITQ: 16-29), median SOFA score was 8 (ITQ: 4-11) and median TISS 28 was 27 (ITQ: 21-30). The median time that patients were observed was 3 (ITQ: 2-6) days, after this period of time patients were either admitted to the ICU or the request for an ICU bed was cancelled. Median total cost per patient treatment was R$12,846.29 (ITQ: R$8279.00-21,763.61), and median daily cost was R$3496.80 (ITQ: R$2668.84-4391.01). Total costs for nonsurvivor patients were higher (R$13,307.08) compared with survivors (R$10,835.53, *p *= 0.041). Hospital mortality was 66.1%.

## Conclusion

Treatment of critically ill patients outside the ICU is costly and associated with poor prognosis. Direct costs of treatment of critically ill patients outside the ICU are higher in nonsurvivors.

